# Long-term *in situ* air quality assessment in closed environments: A gas chromatography–ion mobility spectrometry applicability study

**DOI:** 10.1177/14690667231187502

**Published:** 2023-07-13

**Authors:** Pedro Catalão Moura, Valentina Vassilenko

**Affiliations:** 1 Laboratory for Instrumentation, Biomedical Engineering and Radiation Physics (LibPhys-UNL), Department of Physics, 119482 NOVA School of Science and Technology, NOVA University of Lisbon, Caparica, Portugal; 2 NMT, S.A., Edifício Madan Parque, Rua dos Inventores, Caparica, Portugal

**Keywords:** Indoor air, air quality assessment, closed environments, volatile organic compounds, VOCs, ion mobility spectrometry, gas chromatography

## Abstract

Contemporary life is mostly spent in indoor spaces like private houses, workplaces, vehicles and public facilities. Nonetheless, the air quality in these closed environments is often poor which leads to people being exposed to a vast range of toxic and hazardous compounds. Volatile organic compounds (VOCs) are among the main factors responsible for the lack of air quality in closed spaces and, in addition, some of them are particularly hazardous to the human organism. Considering this fact, we conducted daily *in situ* air analyses over 1 year using a gas chromatography–ion mobility spectrometry (GC-IMS) device in an indoor location. The obtained results show that 10 VOCs were consistently present in the indoor air throughout the entire year, making them particularly important for controlling air quality. All of these compounds were successfully identified, namely acetic acid, acetone, benzene, butanol, ethanol, isobutanol, propanoic acid, propanol, 2-propanol and tert-butyl methyl ether. The behaviour of the total VOCs (tVOCs) intensity during the period of analysis and the relative variation between consecutive months were studied. It was observed that the overall trend of tVOCs closely mirrored the variation of air temperature throughout the year suggesting their strong correlation. The results obtained from this study demonstrate the high quality and relevance of the data, highlighting the suitability of GC-IMS for *in situ* long-term air quality assessment in indoor environments and, consequently, for identifying potential health risks for the human organism in both short-term and long-term exposure scenarios.

## Introduction

Nowadays, most modern life and social interactions occur in closed spaces and indoor environments like work locations, homes, cars and transports, stores, and many other private and public facilities. In fact, this predominance of time spent indoors has been addressed and statistically described in several scientific studies. Klepeis et al.,^1^ for instance, developed a statistical study whose results showed that the survey respondents spend 87% of their daily time in some kind of indoor location. Brasche et al.,^2^ in their turn, were able to conclude that German citizens spend 15.7 h per day in indoor environments. Similar values were registered by the authors for the United States (15.6 h per day) and Canada (15.8 h per day), which means that the average percentage of time spent in indoor spaces is more than 65% of the daytime. The addressed results were corroborated by Schweizer et al., in a recent study. The authors gathered a cohort of 1427 volunteers from 7 different countries and assessed the time spent in indoor areas by all the participants. They were able to determine that the average time spent in indoor locations ranged from 13.5 to 15.8 h per day, meaning that the citizens spend between 56% to 66% of their daily time in the interior of some kind of building, transportation, work location, or public facilities.^3^ Since most of our life is spent indoors, a truly relevant issue arises which must be carefully deemed: indoor air quality.

The degradation of indoor conditions and the lack of air quality are triggered by several known causes. Chemical compounds like carbon monoxide (CO), sulphur dioxide (SO_2_), nitrous oxide (N_2_O) and volatile organic compounds (VOCs), for example, are among the main contributing factors to the loss of air conditions in indoor locations.^4–6^ The presence of these polluting compounds, especially, at hazardous concentration levels, originates the well-known syndrome of sick buildings and, consequently, causes a vast range of pathologies and health conditions in the human organism.^7–9^

One of the hazardous groups of analytes aforementioned, the VOCs, deserves additional attention due to some idiosyncrasies that make them especially dangerous. VOCs are usually released into the environmental air by all kinds of daily-use objects and activities. Building materials, paintings, personal care products, cleaning products, food and cooking, and smoking are very ordinary sources of VOCs.^10–12^ As a consequence, these analytes are present in any kind of indoor location, namely, private houses, schools, hospitals, hotels, stores, industrial factories, airports and stations, and many other closed spaces.^13–15^ In addition, VOCs are volatile at room temperature so, they can effortlessly interact with the human organism and traverse biological structures like pulmonary, cutaneous, and ocular tissues. These interactions lead to processes of mutation by oxidative stress in the human cells and, consequently, to a wide range of health conditions and pathologies.^16,17^ This range includes simpler conditions like allergies, skin irritation and pruritus, but also includes several more complex pathologies like asthma, chronic obstructive pulmonary disease, and a few other inflammatory and respiratory conditions.^18–20^ Some VOCs have even been identified as carcinogenic. Benzene, toluene, ethylbenzene, xylenes and formaldehyde, for example, have been deeply studied regarding their toxicity and direct responsibility in the development of severe forms of lung, oral, breast and gastric cancers.^21–23^

Considering all the addressed facts, the indoor air quality assessment and the *in situ* control of volatile organic compounds in closed habitats are topics of special relevance to the environment and, to public and occupational health.

Several analytical technologies and procedures have been largely employed for the detection, identification and quantification of VOCs. Chromatographic and spectrometry techniques like gas chromatography, infra-red spectroscopy and mass spectrometry are among the most often employed separation techniques.^24,25^ In a different approach, multi-sensor array-based systems have equally been deeply studied regarding their suitability for the complete assessment of VOCs.^26,27^ Nonetheless, all the mentioned techniques and procedures lack some features that are important in the field of VOCs assessment in the indoor air of closed environments, namely, the lack of portability, which hinders *in situ* analyses; the necessity of additional preparation of the sample, which increases the complexity of the analyses; the lower resolution power, sensitivity and selectivity, that inhibit the detection of VOCs at trace levels of concentration; the incapacity for real-time measurement, which delays the entire analysis; the necessity of trained personal due to the instrumental complexity, and several other limitations.^24,28,29^

An analytical technique that has gained significant recognition in the detection, identification and quantification of VOCs is ion mobility spectrometry (IMS). IMS has been extensively investigated in various applications owing to its high sensitivity (typically the detection limits are in the low ppb_v_ or even ppt_v_ ranges of concentration), analytical flexibility and simplicity.^30,31^ This technology was employed during the study here described.

An IMS device allows the separation and consequent identification of each analyte existent in the sample under analysis based on their ion mobility constant, *K*.^31^

(1) $$K=\frac{v_{d}}{E}$$

The original volatile sample is ionised at the beginning of the IMS measurement and then, exposed to a weak homogeneous electric field, *E*, allowing the ions to drift throughout the IMS tube and gain an ion-specific velocity, commonly named as drift velocity, *v_d_*. The ratio between these two values leads to the ion mobility constant, which is also an ion-specific value (equation (1)).^24,30^

Since the velocity corresponds to the quotient between the length of the drift tube, *L*, and the drift time, *D_t_*, required to cross that distance, then, a new formulation for *K* can be expressed as:

(2) $$K=\frac{L}{E.D_{t}}$$

*K* is an ion-specific constant, however, it depends on the specific conditions of pressure, *P*, and temperature, *T*, existent at the time of the measurement so, it is appropriate to normalise *K* to standard values of pressure, *P*_0_ = 760 Torr, and temperature, *T*_0_ = 273.15 Kelvin. It is important to state that the normalisation of the ion mobility constants through the Langevin rule is not as straightforward as expressed since its application is only accurate for zero-size ions and some deviations can occur if applied to cluster ions. The normalised ion mobility constant, *K*_0_, can be written as follows.^24,30,32^

(3) $$K_{0}=K.\frac{P}{P_{0}}.\frac{T_{0}}{T}$$

Since *K*_0_ is also an ion-specific constant, it enables to accurately identify all the ionised analytes originally existent in the sample under analysis.

In order to deal with different levels of humidity of the samples and to increase the specificity of the analysis of complex matrices, the outstanding sensitivity, instrumental simplicity, and almost real-time monitoring capacity of IMS can be improved by a pre-separation procedure. Under the scope of this study, a tandem technology of gas chromatography–IMS (GC-IMS) was employed resulting in a hybrid device with overall improved characteristics for the *in situ* detection of VOCs in air analyses.^31,33^

**Figure 1.**
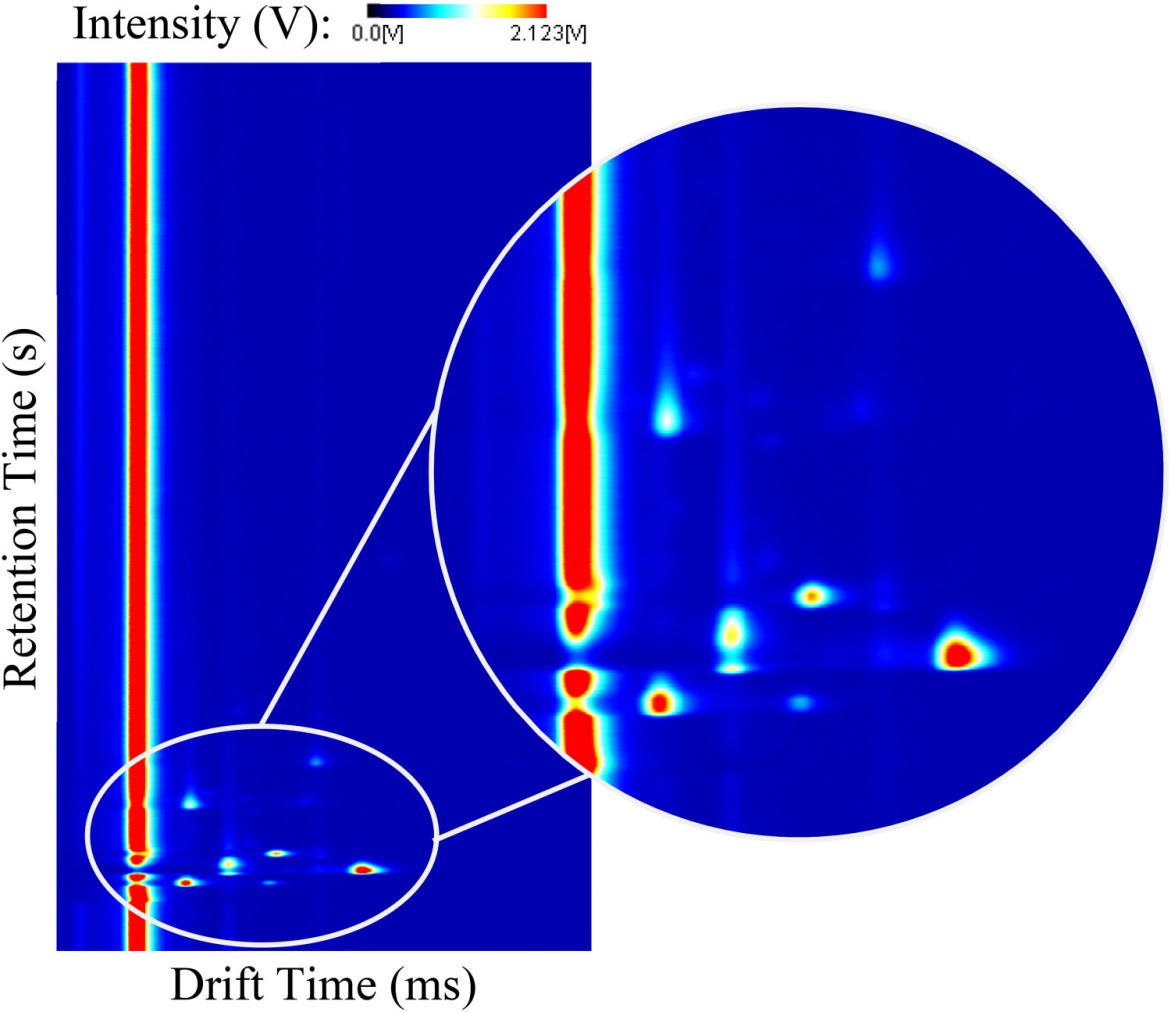
GC-IMS three-dimensional spectrum. GC: gas chromatography; IMS: ion mobility spectrometry.

A detailed description of the operating principle of GC-IMS can be found elsewhere,^15,30^ nonetheless, it is important to state that the GC column acts as a pre-separation procedure in which the analytes existent in the sample are separated by their capacity of adsorption to the internal walls of the chromatographic column. Here, each kind of analyte requires a specific time to traverse the entire column, commonly defined as retention time, *R_t_*. After this, the analytes are ionised and undergo the IMS separation procedure in the drifting of the ions and consequent detection at their specific drift times.^31,34^ As a result, a three-dimensional spectrum is obtained with the compound-specific coordinates corresponding to the retention time (seconds), drift time (milliseconds) and intensity (volts). An arbitrary GC-IMS spectrum with a magnified zone is included in Figure 1. The registered intensity is related to the concentration of each analyte in the original sample and is commonly represented by a colour scale. While the retention and drift times are used for identification purposes, the intensity can be used for quantification purposes.^24,35^

The present study intends to assess the suitability of GC-IMS for *in situ* long-term measurements of VOCs and consequent control of indoor air quality in closed spaces. For this purpose, a year-long study was developed aiming to obtain the GC-IMS spectra of volatile organic compounds, identify the detected analytes and perform the evaluation of the daily variation of the total VOCs (tVOCs) intensity across the period of measurements. It is expected that the achieved results enable authors to assess the suitability of GC-IMS for the aforementioned tasks, to have a deeper understanding of the main analytes continuously present in the considered closed space and of their overall behaviour throughout an extended period, and to test the developed procedure for future detection of potentially hazardous VOCs relevant for the public health.

## Materials and methods

### Air samples

Aiming to evaluate the indoor air quality of a closed space for a long period, the GC-IMS device was assembled in a specific room whose main purpose was to simulate a generic closed space environment. The air samples were automatically collected from the room air by the device's pump without requiring the operator's intervention. Daily air measurements were meticulously performed at the same time across the study period. The temperature and humidity conditions of the room were not artificially controlled during the entire study, that is, no heating, cooling, humidifier or dehumidifier devices were used. The environmental humidity in the room varied between 40% and 60%, mainly depending on the month of the year. The affluence consisted of five people working in the interior of the room for 8 h per day, 5 days per week. In this way, all the potential sources of VOCs, affluence of people, social interactions and work regime were kept unaltered.

*In situ* measurements of the indoor air were performed on a daily regime between September 2019 and July 2020. No measurements were performed during the weekends. Due to an unexpected period of quarantine period provoked by the COVID-19 pandemic, it was not possible to perform air analyses between 16 March and 15 May 2020. It was equally impossible to perform air analyses on several holidays across the 11 months and the weekends. August was excluded due to the vacation period of the facilities. Regarding all the limitations, a total of 167 measurements were considered (September – 21 measurements, October – 21 measurements, November – 18 measurements, December – 15 measurements, January – 15 measurements, February – 18 measurements, March – 10 measurements, April – 0 measurements (disregarded), May – 10 measurements, June – 19 measurements and July – 20 measurements). The final and complete plan of measurements is represented in Table 1, included in the appendix.

**Table 1.** Calendar of all the measurements performed during the 11 months of study.

Days	Months										
	2019				2020						
	Sept.	Oct.	Nov.	Dec.	Jan.	Feb.	Mar.	Apr.	May	Jun.	Jul.
1		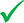	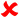		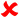			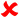	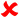	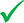	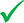
2	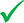	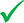		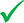	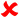		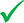	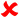		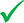	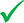
3	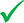	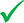		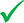	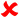	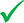	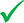	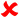		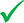	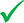
4	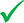	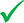	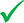	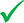		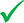	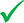		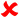	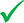	
5	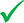		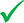	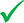		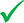	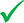		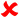	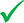	
6	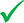		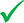	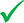	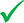	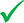	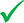	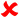	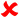		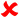
7		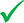	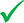		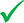	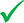		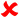	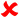		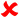
8		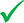	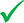		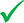			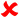	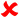	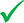	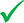
9	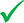	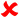		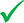	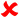		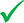	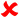		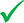	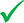
10	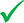	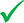		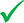	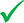	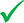	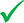	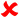		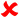	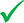
11	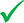	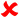	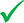	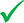		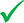	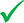		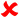	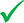	
12	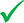		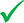	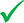		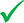	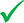		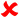	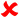	
13	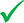		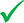	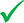	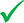	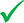	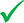	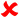	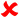		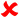
14		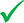	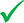		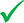	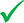		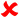	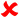		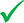
15		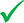	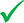		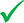			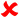	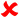	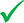	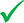
16	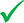	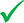		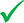	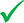		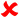	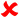		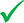	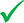
17	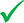	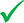		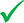	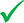	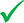	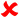	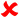		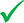	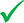
18	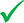	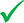	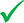	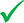		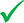	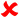		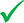	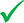	
19	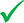		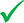	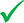		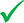	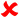		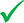	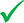	
20	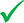		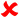	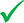	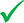	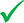	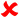	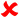	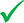		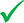
21		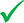	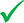		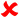	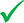		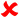	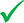		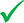
22		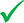	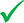		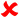			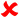	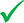	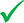	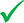
23	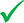	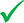		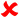	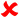		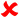	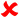		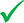	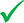
24	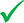	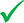		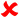	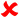	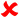	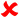	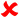		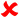	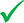
25	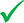	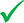	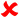	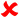		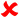	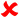		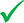	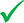	
26	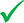		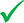	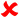		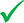	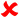		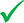	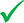	
27	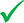		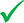	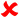	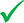	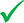	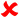	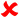	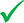		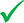
28		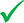	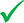		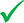	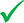		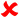	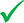		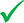
29		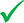	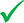		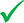			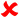	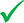	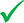	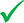
30	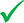	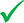		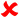	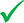	–	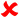	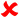		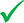	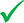
31	–	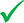	–	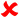	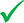	–	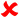	–		–	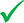

### Gas chromatography–Ion mobility spectrometry

All experimental measurements were performed with a GC-IMS apparatus produced by G.A.S. Dortmund (Germany). A GC column with 30 m of length and 0.53 mm of internal diameter coated with stainless steel with a mid-polar stationary phase of trifluoropropylmethyl polysiloxane with a thickness of 1 μm was assembled in the device (MXT-200 model). The IMS section of the device was equipped with a 300 MBq ionisation source of Tritium (3H–β radiation), with a 98 mm length drift tube assembled with an electric field of 500 V/cm of strength and a 5 kV switchable polarity. The operating parameters used in the GC-IMS device for the present measurements are summarised in Table 2.

**Table 2.** Operating parameters of the GC-IMS device.

Parameters	Values	Units
Sample Loop Volume	1	mL
GC Column Model	MXT-200	–
GC Column Length	30	m
GC Column Diameter	0.53	mm
GC Temperature	343.15	Kelvin
Gas Nature	Purified Air	–
Carrier Gas Flow	10	mL/min
Drift Gas Flow	150	mL/min
Ionisation Source	Tritium – β Radiation	–
Ionisation Intensity	300	MBq
Ionisation Polarity	Positive	–
Drift Region Length	9.8	cm
Drift Potential Difference	5	KV
IMS Temperature Range	297.15–301.15	Kelvin
IMS Pressure Range	757–760	Torr
Electric Field Intensity	500	V/cm
Resolving Power Range	65–70	–

GC: gas chromatography; IMS: ion mobility spectrometry.

### VOCs assessment

From each measurement, a three-dimensional spectrum was plotted and exported with the Laboratory Analytical Viewer software, version 2.2.1., from the device manufacturer, for further processing. A total of 167 spectra from the room air analyses were processed by the abovementioned software to obtain the drift and retention times, as well as, the intensity for every detected analyte.

The drift and retention times were used for identification purposes by crosschecking the values of the detected analytes with a previously developed database of volatile organic compounds. Additional details on the database development are given elsewhere.^15^ Nonetheless, it is relevant to mention that chemical standards samples were analysed with the same device and operating mode, and their respective values were registered in the database. By crosschecking the values of the database with the values registered during the *in situ* analyses, accurate identification of the VOCs was achieved.

The intensity of each analyte detected was statistically processed for several purposes. Considering only the VOCs present in the air samples throughout the entire study, their specific intensities were summed, and the normalised total intensity of VOCs was calculated. Then, this value was used to assess the repeatability and, consequently, the statistical relevancy of the GC-IMS data. The relative variation of the tVOCs intensity between consecutive months was equally calculated and plotted in a graph. As mentioned, April was disregarded from all the data processing due to the inexistence of measurements.

## Results and discussion

### VOCs identification

As previously described, the identification of the detected analytes was achieved by crosschecking their drift and retention times with the times registered in a predeveloped database of VOCs. For purposes of identification, only the long-lasting compounds were considered, therefore, all the analytes that were not present during all the 11 months were disregarded.

A total of 16 analytes were accurately identified with the library and, considering monomers, dimers and even trimers of the same compound, the 16 analytes totalise 10 VOCs. Among these 10, five belong to the organic family of alcohols (ethanol, isopropanol, propanol, isobutanol and butanol), two are carboxylic acids (acetic acid and propanoic acid), commonly known as fatty acids, one ether (tert-butyl methyl ether), one ketone (acetone), and one aromatic hydrocarbon (benzene). Table 3 includes all the identified VOCs, their respective retention and drift times, normalised ion mobility constants, and CAS numbers.

**Table 3.** Identified VOCs and respective retention and drift times, normalised ion mobility constants, and CAS numbers.

#	VOCs	Note	Retention time (*s*)	Drift time (*ms*)	Drift time (*RIP relative*)	$K_{0}$ $\left(cm^{2}/V.s\right)$	CAS number
1	Ethanol	Monomer	73	7.74	1.06	1.95	64-17-5
2		Dimer		8.44	1.15	1.79	
3	Isopropanol	Monomer	82	8.10	1.10	1.86	67-63-0
4		Dimer		8.84	1.21	1.71	
5		Trimer		9.20	1.25	1.64	
6	Tert-butyl Methyl ether	Monomer	90	8.50	1.16	1.78	1634-04-4
7		Dimer		9.56	1.30	1.58	
8		Trimer		10.34	1.41	1.46	
9	Acetone	Monomer	91	8.85	1.21	1.71	67-64-1
10	Propanol	Monomer	96	8.27	1.13	1.83	71-23-8
11	Isobutanol	Monomer	118	8.72	1.19	1.73	78-83-1
12		Dimer		10.28	1.40	1.47	
13	Acetic acid	Monomer	118	7.78	1.06	1.94	64-19-7
14	Benzene	Monomer	120	8.23	1.12	1.83	71-43-2
15	Butanol	Monomer	141	8.83	1.20	1.71	71-36-3
16	Propanoic acid	Monomer	168	8.19	1.12	1.84	79-09-4

VOC: volatile organic compound; CAS: chemical abstract service; RIP: reactant ion peak.

Alcohol-based VOCs are known for their hazardousness not only to the environment but mainly to the human organism, being eyes, skin and throat irritation the most common reactions. Nonetheless, more complex reactions to alcohol-based compounds exposure have been reported, namely, narcosis and central nervous system depression. Due to these facts, most alcohol-based solutions, like coatings, inks or personal care products, have even been replaced by considerably safer water-based versions.^36,37^

Severe eyes and skin irritation are equally common reactions of the human organism if exposed to fatty acids like acetic and propanoic acid, ketones or ethers. Dizziness, nausea, suffocation, vomiting and hyperaemia have been reported in scenarios of long-term exposure to all these analytes.^36,38,39^

Finally, the aromatic hydrocarbon detected in the indoor air samples of the closed space analysed is known for its particular hazardousness. Vulgarly known as BTEX compounds, benzene, toluene, ethylbenzene and xylenes have been largely studied and the risks to human health in both short-term and long-term exposures are well-known.^40,41^

Considering all the mentioned facts about the dangerousness of VOCs to the human organism and since these 10 compounds are always present throughout the entire year, all of them deserve special attention and must be of special interest for the maintenance of air quality in a closed environment and for the assessment of exposure conditions of the workers who frequently use the space. Further studies are required to assess the original sources responsible for the emission of the analytes.

### Repeatability

**Figure 2.**
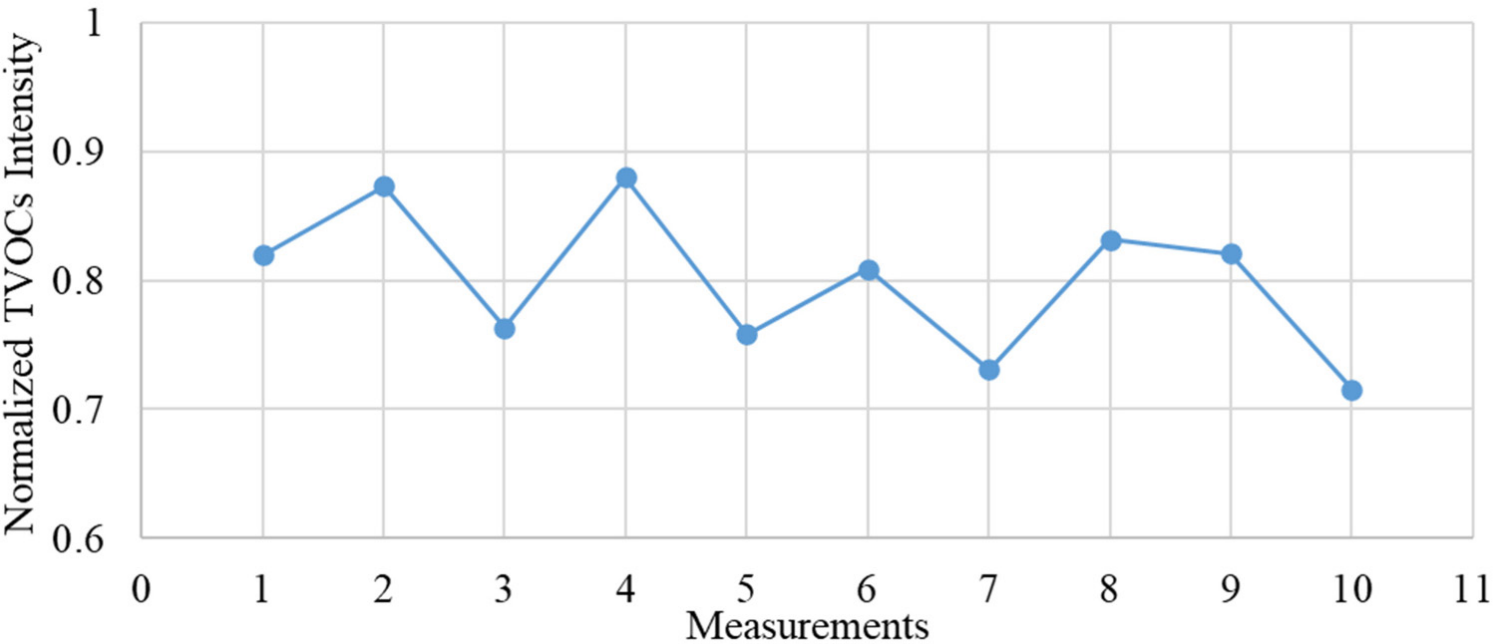
Variability line chart for the GC-IMS measurements. The average value for the normalised tVOCs intensity and the respective average deviation are (0.80 ± 0.05). GC: gas chromatography; IMS: ion mobility spectrometry; tVOC: total volatile organic compound.

In order to assess the repeatability and, consequently, the precision of measurements and quality of the proposed method, the statistical relevancy of the data collected with the GC-IMS device and the variability of the intensity of the analytes were analysed. For this purpose, the normalised tVOCs intensity registered for 10 subsequent analyses was represented in a line graph. Figure 2 illustrates the repeatability achieved with the GC-IMS technology. The average value of the normalised intensity and the respective average deviation achieved during the processing of this dataset were (0.80 ± 0.05). These values prove that not only the GC-IMS ensures outstanding repeatability of the collected data but also, that this technology is more than suitable for *in situ* long-term air quality control in closed environments.

### tVOCs variation

As described, the total VOCs intensity was determined and normalised for all 11 months. Aiming to understand the evolution of the tVOCs value throughout the entire study, the relative variation between consecutive months was equally calculated. To do so, the percentual variation of tVOCs, for each month, was calculated relatively to the previous month. Table 4 summarises the normalised tVOCs intensity and the relative variation in percentage form. Information elucidating if the variation corresponds to an increase or decrease in the tVOCs intensity is also included.

**Table 4.** Normalised tVOCs intensity and respective variation (%) between consecutive months.

Year	Month	Normalised tVOCs intensity	Relative variation (%)	Increase or decrease (↑ or ↓)
2019	September	0.93	–	–
	October	0.87	6.43	**↓**
	November	0.76	13.61	**↓**
	December	0.70	7.21	**↓**
2020	January	0.55	22.08	**↓**
	February	0.71	30.19	**↑**
	March	0.82	15.25	**↑**
	May	0.88	7.23	**↑**
	June	0.92	4.37	**↑**
	July	0.99	7.44	**↑**

tVOC: total volatile organic compound.

Considering the results represented in the previous table, it is possible to remark on the evident decrease in the tVOCs intensity between September 2019 and January 2020. From February to July 2020, data reports a successive increase in the total average intensity. Coincidently, this behaviour is similar to the variation of the average air temperature of the country where the analysed closed spaced is located. The air temperature tends to decrease between September and January and tends to increase from January onwards. Not being the only impacting factor, air temperature plays a truly relevant role in the volatilisation of VOCs, as addressed previously.

**Figure 3.**
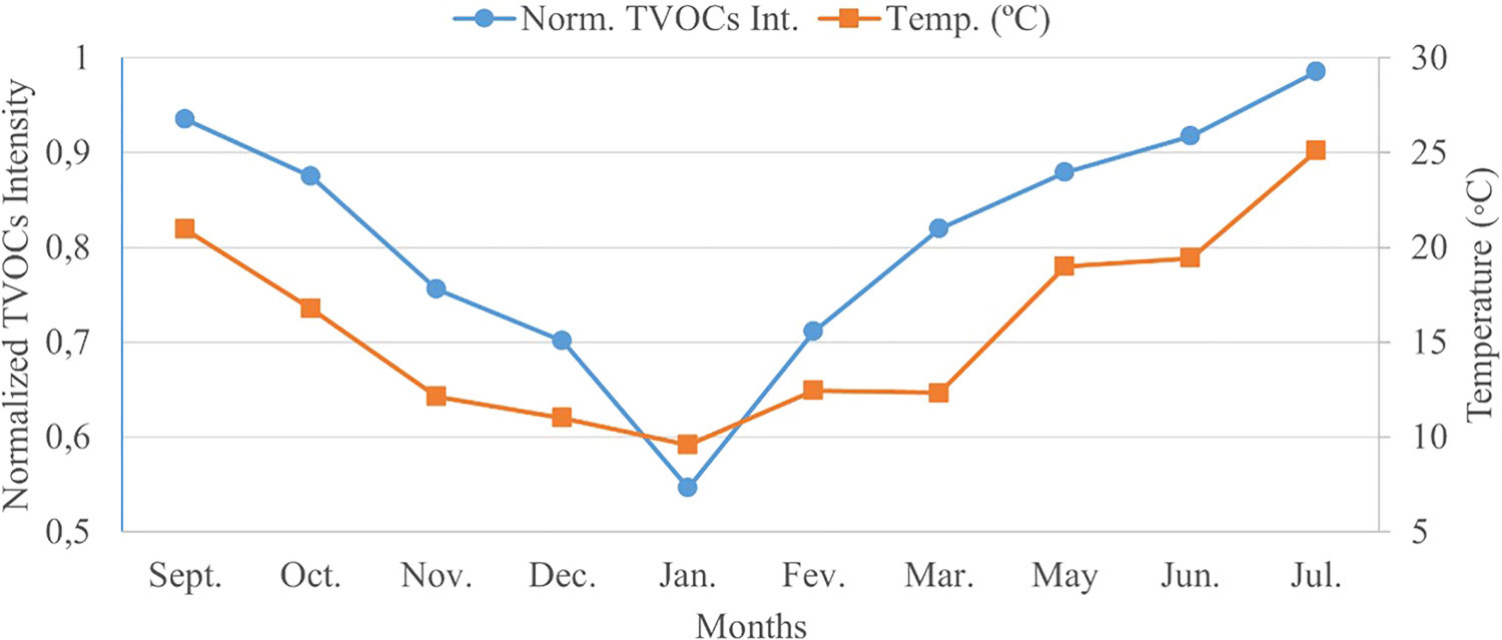
Variation of the total average intensity of VOCs between September 2019 and July 2020 (April was disregarded) versus the temperature variation. VOC: volatile organic compound.

To easily qualify the variation of the average intensity along the considered months, a graph was plotted. The graph is represented in Figure 3, where the months are denoted on the x-axis and the left y-axis corresponds to the normalised tVOCs intensity. The average air temperature per month, as recorded by the national institute of meteorology,^42^ is denoted on the right y-axis.

As mentioned, the graph of Figure 3 mimics the behaviour of the tVOCs intensity throughout the 11 analysed months. As included in Table 4, the normalised tVOCs intensity experiences a decrease between September and January. Coincidently, these months typically see a decrease in the overall weather temperature, hindering the volatilisation of the VOCs. On the other side, between January and July, the total intensity of VOCs continuously increases. This can be justified by the coincident increase in air temperature during these months, easing the volatilisation of the analytes. This correlation can be speculated since no other alterations, affluence variation or re-placement of potential sources of VOCs were made in the closed spaced studied throughout the year.

**Figure 4.**
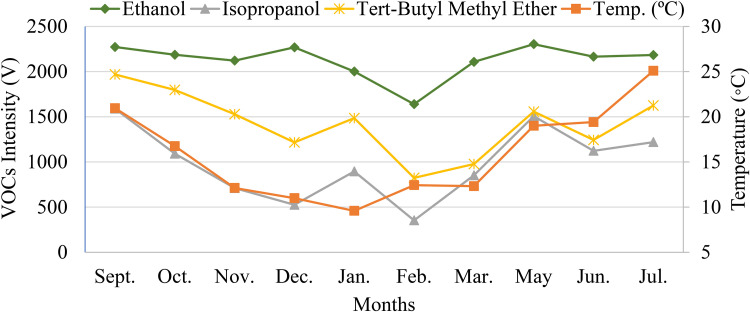
Variation of the intensity of ethanol, isopropanol and tert-butyl methyl ether between September 2019 and July 2020 (April was disregarded) versus the temperature variation.

In order to assess if the behaviour of the intensity variation for individual VOCs throughout the studied months also mimics the temperature variation, the absolute intensity (V) per month of each analyte was plotted against the temperature graph. Three of these graphs are illustrated in Figure 4, namely, the graph of intensity variation for ethanol, isopropanol and tert-butyl methyl ether.

It is evident that all these three VOCs exhibit behaviours that mimic the variation of the air temperature throughout the entire year. Similar results were attained for all the remaining VOCs, that is, all the 10 considered analytes seemed to exhibit intensity variations comparable with the temperature variation. Markedly, further studies should be made in order to assess the correct factors inducing the variation of the tVOCs intensity, identify the main sources of VOCs and, consequently, properly evaluate the impacts on human health.

## Conclusions

Aiming to assess the suitability of GC-IMS for *in situ* long-term air quality control in closed environments and, consequently, evaluate the potential risks to the human organism, a year-long case study was established. From the measurements undertaken on a daily regime, a total of 167 spectra were statistically processed and all the analytes present in the air samples throughout the entire year were considered for the study. Ten analytes were accurately identified with a predeveloped database of VOCs, namely ethanol, 2-propanol, tert-butyl methyl ether, acetone, propanol, isobutanol, acetic acid, benzene, butanol and propanoic acid. All these analytes are well-studied regarding their hazardousness and risks to human health.

A repeatability graph was plotted considering the normalised tVOCs intensity of 10 consecutive measurements. An average tVOCs value of 0.80 and an average deviation of 0.05 prove the good precision of the measurements and confirm the quality control of the method for air quality analyses. The behaviour of the tVOCs throughout the entire year was assessed, and the relative variation between consecutive months was calculated. A decrease in the tVOCs intensity was registered between the months and September and January, matching the behaviour of the overall air temperature. From February forward, the tVOCs intensity registered a constant increase, again, mimicking the air temperature provided by the national weather institute. Since the VOCs are volatile at room temperature, their volatilisation is especially affected by the variation in air temperature. This direct relationship was evident in the results achieved during the study, that is, months with higher air temperature led to higher concentration levels of VOCs and, consequently, to increased exposure risks to human health.

Overall, this 1-year-long case study confirmed the necessity of air quality control in indoor, closed and highly populated environments. It was established that, due to its characteristics, namely, its outstanding sensitivity, very low concentration range, good precision, instrumental simplicity, almost-real-time measuring capacity and portability, GC-IMS is completely suitable for *in situ* long-term VOCs detection and air quality control in closed environments. Further studies must be carried on to accurately assess the VOCs concentration, identify the main sources of emission, and evaluate the direct consequences to human health in both short-term and long-term scenarios.

## Appendix

see Table 1.
